# Clinical validation of an artificial intelligence-based diabetic retinopathy screening tool for a national health system

**DOI:** 10.1038/s41433-020-01366-0

**Published:** 2021-01-11

**Authors:** José Tomás Arenas-Cavalli, Ignacio Abarca, Maximiliano Rojas-Contreras, Fernando Bernuy, Rodrigo Donoso

**Affiliations:** 1 TeleDx, Santiago, Chile; 2 Department of Ophthalmology, Universidad de Chile, Santiago, Chile

**Keywords:** Public health, Retinal diseases

## Abstract

**Objective:**

To evaluate the accuracy and validity of an automated diabetic retinopathy (DR) screening tool (DART, TeleDx, Santiago, Chile) that uses artificial intelligence to analyze ocular fundus photographs for potential implementation in the national Chilean DR screening programme.

**Method:**

This was an observational study of 1123 diabetic eye exams using a validation protocol designed by the commission of the Chilean Ministry of Health personnel and retina specialists.

**Results:**

Receiver operating characteristic (ROC) analysis indicated a sensitivity of 94.6% (95% CI: 90.9–96.9%), specificity of 74.3% (95% CI: 73.3–75%), and negative predictive value of 98.1% (95% CI: 96.8–98.9%) for the automated tool at the optimal operating point for DR screening. The area under the ROC curve was 0.915.

**Conclusions:**

The results of this study suggest that DART is a valid tool that could be implemented in a heterogeneous health network such as the Chilean system.

## Introduction

Given the rapidly increasing prevalence of diabetes mellitus (DM) in the Chilean and world populations [1], rates of DM-related vision loss and blindness are rising considerably [2]. There is a pressing need to enhance the detection and prevention of diabetic retinopathy (DR), but significant gaps in the availability of ophthalmological resources for DM patients present a major obstacle.

The World Health Organization defines screening as “the presumptive identification of unrecognized disease or defect by the application of tests, examinations, or other procedures which can be applied rapidly. Screening tests sort out apparently well persons who probably have a disease from those who probably do not. A screening test is not intended to be diagnostic” [3, 4].

Importantly, it is widely accepted that screening tests are not diagnostic tests but rather are procedures for identifying high-risk patients who should be referred for specialist evaluation. Therefore, there may be a role for nonmedical professionals, automated systems, and telemedicine in DR screening [5].

In the context of DR, the International Council of Ophthalmology (ICO) emphasizes that “there is currently a significant shortfall of ophthalmologists in developing countries,” stating that “it is necessary to aggressively train eye care teams now to alleviate the current and anticipated deficit of ophthalmologists worldwide” [6]. In Latin America, guidelines based on recent experimental findings and clinical experience have been published to help “ensure that patients receive the best available care in a timely manner” [7].

The ICO notes that “screening for DR is an important aspect of DM management worldwide. Even if an adequate number of ophthalmologists are available, using ophthalmologists or retinal subspecialists to screen every person with DM is an inefficient use of resources” [8]. The urgency of increasing DR screening coverage and the need to overcome various technical obstacles associated with current screening methods have led to the development and validation of technologies that can automatically recognize signs of DR in fundus photographs [9–12]. These new methods can separate populations that require a specialist referral from those that do not while reducing the number of DR screening cases that must be evaluated by an ophthalmologist.

Moreover, the Joint Declaration from the International Diabetes Federation, ICO, World Council of Optometry, and International Agency for the Prevention of Blindness recommends increasing “investment in the development of more cost-effective, sturdy and automated technologies for assessment, referral, and treatment” [13]. In summary, the Chilean (and, most likely, every other Latin American) public health system needs a technological solution that would improve the efficiency of interpreting preventive ocular exams and optimize the use of ophthalmological resources.

In the Chilean healthcare network, multiple guaranteed entry points exist for cardiovascular and DM risk populations to be identified, namely, through morbidity consultation at primary care and annual preventive exams for adults and the elderly. Encouraged by performance goals and allocated per-capita budgets, centralized control is carried out to identify people with DM in the community, achieving a degree of awareness of their condition of 85% among patients [14], above the global average of 50% [1].

The same policies and incentives apply to DR screening and treatment. To address referred patients, there is a national network of 29 public health services [15] with their respective ophthalmology departments in secondary and tertiary centres (57 across the country [16]). The resolution rate was not found to be properly documented, but DR screening coverage on the primary care network was reported at 32.4% in 2016 [17].

This study compares the performance of an artificial intelligence (AI)-based DR screening system called DART (TeleDx, Santiago, Chile) [18–20] with an assessment performed remotely by a clinical ophthalmologist [21], using retinal images acquired according to the EURODIAB protocol [22]. The objective of the study was to determine whether the system is sufficiently valid as a screening method for its application in the Chilean public health network.

## Materials and methods

An observational study was conducted to evaluate the accuracy and validity of DART for potential implementation in the Chilean public DR screening programme. The study was designed to fulfill the requirements of a validation protocol designed by a commission of specialists working with the Chilean Ministry of Health (Minsal, for its acronym in Spanish).

This validation study was retrospective and observational (noninvasive). It was carried out by reviewing the ocular fundus photographs of patients included in the sample. No patient was approached directly regarding the study, and patient care was not affected in any way by it. Only anonymous or pseudoanonymous data were used after collection as part of routine clinical care. Codes were assigned to the episodes for reference during data processing and analysis. Patient information was accessed according to all relevant standards of privacy and confidentiality at all stages of data collection and analysis. The study was carried out using a double-blind methodology for the ophthalmologists and the AI tool. Careful measures were taken to protect the security of the data and to avoid releasing personal health information to any person outside the research team or to non-clinical personnel on the project, complying with Minsal regulations regarding confidentiality and protection of sensitive data (user authentication, personnel contracts, protection of computer servers, etc.).

Minsal approved this study and deemed that patient informed consent was not necessary given the retrospective nature of the study and given the de-identification of all patient data, consistent with prior institutional review boards’ determinations for similar protocols [23]. Approval was also obtained from the ethics committee of the East Metropolitan Health Service, and agreements were established with all institutions involved. The institutional ethics committee also determined that no informed consent was needed due to the anonymous nature of the study data and the fact that no procedure related to the study would affect patient care, consistent with relevant precedents from international literature [12, 23–25]. The study was conducted in agreement with the principles of the Declaration of Helsinki [26].

A major requirement of the validation protocol was the inclusion of a representative sample of cases (exams), both quantitatively and qualitatively, to ensure accurate characterization of the population living with DM in Chile. The sample was selected from five community centres catering to the primary care of the Chilean population in five different communes to provide inclusive coverage of the nation’s geographic regions to reflect the unique population features of each area and to ensure that the findings would adequately represent the various diabetic ophthalmological screening programmes throughout the country.

The inclusion criteria for sample selection were ocular fundus photographs performed on a patient with DM during the data collection period in one of the five participating communes. To minimize bias, cases meeting inclusion criteria were selected chronologically; the first two of every five exams performed in each participating commune during the data collection period were selected. The data were collected from November 11, 2014 to December 18, 2016.

This AI tool relies on precise detection of abnormalities in ocular fundus images using two fully convolutional neural networks [27]: one for detecting signs of diabetic macular edema (DME) and the other for detecting signs of DR (in particular, vascular lesions or abnormal vascular signs). Both outputs are then weighted to obtain a probability of DR per eye, and the highest probability between them stays as the final probability. These networks were trained using ocular fundus photographs obtained according to the EURODIAB protocol [22] and analyzed by one ophthalmologist (from a panel of eight).

Each case was processed by the automated system, which provided a dichotomous result: “positive” for any grade of DR (pathology is present) or “negative” (pathology is not present). This dichotomous result was based on a value indicating the degree of membership of each case to one of the two possible states, as calculated by the algorithm. Exams with a score at or above a strict threshold of 0.0166 were classified as positive, and results below this threshold were classified as negative. This threshold was defined using receiver operating characteristic (ROC) curve analysis set at an operating point that provided high sensitivity to minimize the rate of false negatives (FN) during screening.

Exams were retrieved using the national teleophthalmology web platform administered by Minsal. This platform operates under the technical-administrative authority that governs primary care ophthalmology. The process starts when a patient is attended by an ophthalmology technician in a primary care centre, where the professional assesses whether it is necessary or not to dilate the patient’s pupil.

Then, the images are taken using non-mydriatic cameras whose main requirement is to be a desktop camera (not portable), to have at least a 10-megapixel sensor and to capture 45-degree field retinal images. Two retinographies are taken for each eye (temporal and nasal captures), following the EURODIAB protocol [22].

Each retinal image is assessed by an ophthalmologist using a web platform based on the International Clinical Diabetic Retinopathy severity scale [21]. For each eye, the physician rates the severity of DR as follows: no apparent DR (R0), mild non-proliferative DR (R1), moderate non-proliferative DR (R2), severe non-proliferative DR (R3), proliferative DR (R4), or ungradable if the quality of the photograph is inadequate to confirm the suspected grade of DR. Furthermore, the ophthalmologist could indicate suspicion of DME in addition to any DR ranging from R0 to R4. Then, the overall exam grade is defined as the most severe grade between both eyes.

For this analysis, the ophthalmologist gradings were then grouped into two aggregate classifications to produce a dichotomous screening result. A case was classified as “ophthalmologist-negative” for the absence of DR (DR-) if the grading was R0 and there were no other findings, or “ophthalmologist-positive” (DR+) if any other grading was given. In other words, any exam with a grading of R1, R2, R3, R4, or ungradable and/or suspicion of DME was classified as DR+; and a case was classified as DR- only if rated as R0 and not categorized as suspicious for DME.

The results were organized into a 2 × 2 confusion matrix, with the ophthalmologist grading for each case on one axis (DR+, DR-) and the results of the automated AI-based system on the other (positive, negative). The confusion matrix was used to classify the results as true negative (TN), false positive (FP), true negative, or FN. These data were then used for ROC analysis to assess the discriminatory capacity of the system.

The minimum required sample size was calculated using two formulas from the literature for screening tests with dichotomous results (in this case, positive or negative for the presence of DR), one based on sensitivity and the other on specificity. Similar formulas have been used in previous studies on automated technologies for DR screening [12]. The literature suggests setting the sensitivity at 80% for this type of test [28–30], but there is no expert consensus on recommended levels of specificity to date [12, 31]. Furthermore, it has been shown that similar systems with specificities between 20 and 53% are cost-effective alternatives to a purely manual grading of DR [32, 33]. This value was finally set at 50% by the commission of specialists of Minsal. These formulas also take into account the prevalence of the pathology. According to the Pan-American Association of Ophthalmologists, DR prevalence may be as high as 40% within the population living with DM in some countries of the region [5]. However, the most recent Chilean epidemiological study estimates a prevalence of 30% [5]. The formulas are shown below:

1 $$n_{se} = \frac{{Z_{\frac{\alpha }{2}}^2 \ast se \ast \left( {1 - se} \right)}}{{d^2 \ast prev}}$$

2 $$n_{sp} = \frac{{Z_{\frac{\alpha }{2}}^2 \ast sp \ast \left( {1 - sp} \right)}}{{d^2 \ast (1 - prev)}}$$

where: *n*_*se*_: number of patients required for a given sensitivity. *n*_*sp*_: number of patients required for a given specificity. *se*: sensitivity, in this case fixed at 80%. *sp*: specificity, in this case fixed at 50%. $Z_{\frac{\alpha }{2}} \!:\!$ 1.96 for 95% confidence level. *d*: maximum margin of error, in this case 5% for a precision of 95%. *prev*: population level prevalence, in this case fixed at 30% of the population with DM.

As the two formulas produced different minimum sample sizes, the greater value was selected. Thus, given the formula outputs shown below, the minimum sample size was calculated at 820 subjects.

3 $$n_{se} = \frac{{Z_{\frac{\alpha }{2}}^2 \ast se \ast \left( {1 - se} \right)}}{{d^2 \ast prev}} = \frac{{1.96^2 \ast 0.8 \ast (1 - 0.8)}}{{0.05^2 \ast 0.3}} = 819.5 \cong 820$$

4 $$n_{sp} = \frac{{Z_{\frac{\alpha }{2}}^2 \ast sp \ast \left( {1 - sp} \right)}}{{d^2 \ast (1 - prev)}} = \frac{{1.96^2 \ast 0.5 \ast (1 - 0.5)}}{{0.05^2 \ast (1 - 0.3)}} = 548.8 \cong 549$$

## Results

**Fig. 1:**
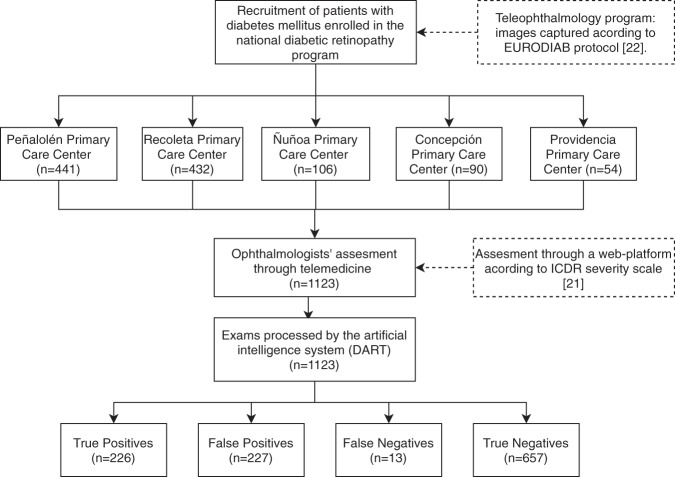
Participants workflow in the study. The bottom section shows the results and allows all the relevant indicators to be computed.

A total of 1123 eye exams from different patients (exceeding the minimum sample size required) followed the flow described in Fig. 1 and were collected across the different communes. Cameras were among the most frequently found in the national screening programme, namely, the AFC-330 (Nidek, Gamagori, Japan) in Peñalolén (441 cases), Recoleta (432 cases), and Ñuñoa (106 cases); the CR-2 (Canon, Tokyo, Japan) in Concepción (90 cases); and the TRC-50ex (Topcon, Tokyo, Japan) in Providencia (54 cases).

Of the total cases, 452 (40.2%) were male, and 671 (59.8%) were female. The average patient age at the time of the exam was 63 years (standard deviation 12.7). The prevalence of exams with R1, R2, R3, R4, or ungradable and/or suspicion DME was 21.3%. The distribution of severity grades in the sample is detailed in Table 1.

**Table 1:** Distribution of DR severity and DME in the sample.

Grade	DME absent	DME present
R0	884	10
R1	21	11
R2	81	58
R3	24	6
R4	5	3
Ungradable	20	–

The results are presented in a 2 × 2 confusion matrix (Table 2) comparing the screening results provided by the AI system with the remotely delivered clinical opinion of the ophthalmologist, followed by summary indicators of the diagnostic capacity of the system as assessed using ROC analysis.

**Table 2:** Screening results based on clinical opinion of ophthalmologist (teleophthalmology) v. AI system.

Test result	Ophthalmologist classification	
	DR+	DR−
Positive	226	227
Negative	13	657
Total	239	884

Based on the results presented in Table 2, indicators defined as critical for the study analysis were calculated, along with their respective confidence intervals (CIs), as shown in Table 3.

**Table 3:** Summary indicators.

Indicator		Value	95% confidence interval	
Name	Formula		Lower bound	Upper bound
Prevalence	${\mathrm{prev}} = \frac{{{\mathrm{TP + FN}}}}{{TN + FN + TP + FP}}$	0.213	0.190	0.238
Sensitivity	${\mathrm{se = }}\frac{{{\mathrm{TP}}}}{{TP + FN}}$	0.946	0.909	0.969
Specificity	${\mathrm{sp}} = {\mathrm{TN/TN + FP}}$	0.743	0.733	0.750
False positive rate	${\mathrm{FPR = 1}} - {\mathrm{sp}}$	0.256	0.250	0.267
False negative rate	${\mathrm{FNR = se}}$	0.054	0.031	0.091
Positive predictive value	${\mathrm{PPV = }}\frac{{{\mathrm{TP}}}}{{{\mathrm{TP + FP}}}}$	0.499	0.480	0.511
Negative predictive value	${\mathrm{NPV = }}\frac{{{\mathrm{TN}}}}{{{\mathrm{TN + FN}}}}$	0.981	0.968	0.989
Positive likelihood ratio	${\mathrm{PLR = }}\frac{{{\mathrm{se}}}}{{{\mathrm{1}} - {\mathrm{sp}}}}$	3.682	3.408	3.87
Negative likelihood ratio	${\mathrm{NLR = }}\frac{{{\mathrm{1}} - {\mathrm{se}}}}{{{\mathrm{sp}}}}$	0.073	0.041	0.124
Diagnostic odds ratio	${\mathrm{DOR = }}\frac{{{\mathrm{TP}} \ast {\mathrm{TN}}}}{{{\mathrm{FP}} \ast {\mathrm{FN}}}}$	50.318	27.456	94.065
Relative risk	${\mathrm{RR = }}\frac{{{\mathrm{PPV}}}}{{{\mathrm{1}} - {\mathrm{NPV}}}}$	25.712	14.768	46.479
Percentage of concordance	${\mathrm{POC = }}\frac{{{\mathrm{TP + TN}}}}{{TN + FN + TP + FP}}$	0.786	0.771	0.796
Test negativity rate	${\mathrm{TNR = }}\frac{{{\mathrm{TN + FN}}}}{{TN + FN + TP + FP}}$	0.597	0.567	0.625
Test positivity rate	${\mathrm{TPR = }}\frac{{{\mathrm{TP + FP}}}}{{TN + FN + TP + FP}}$	0.403	0.374	0.432

**Fig. 2:**
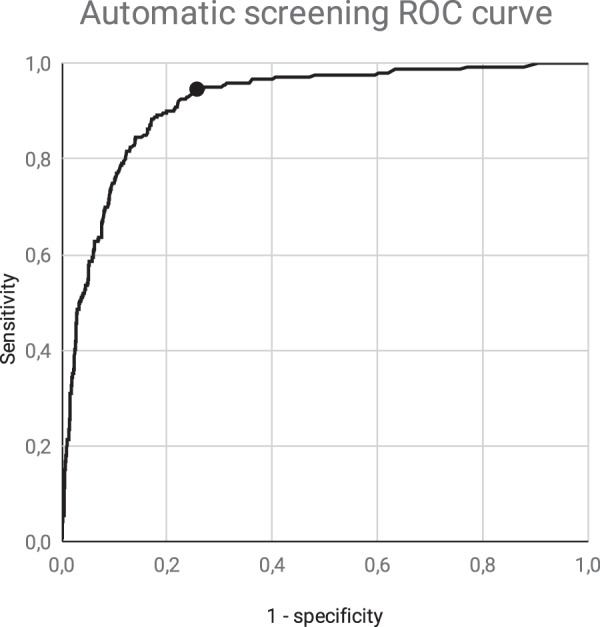
ROC curve showing the sensitivity against the FP rate (1-specificity) of the AI system. The dot represents the selected operating point.

The area under the ROC curve (AUC) shown in Fig. 2 is 0.915. Data regarding operating points along the curve could be used to adjust the model for different needs in terms of cost efficiency to address public healthcare challenges associated with DR in other contexts.

Varying the threshold by as much as ±10% only altered the sensitivity of the test by 0.025 and the specificity by 0.057. Even when the threshold was increased by 100%, test performance remained within the parameters required by the commission (0.841 sensitivity, 0.859 specificity).

The original evaluation (criterion A) categorized an exam as positive if the ophthalmologist grading was R1 or greater and/or suspicion DME or ungradable. Using criterion A, 239 cases were categorized as positive, from a total of 1123. An additional sensitivity analysis using two other criteria (B and C) was then performed.

- Criterion B: Each exam was categorized as positive if the classification was R2 or greater, DME, or ungradable. This criterion was based on typical practice for recommending a specialist referral according to other authors [21]. From the sample (1123), 218 cases were categorized as positive.
- Criterion C: Identical to criterion A, but ungradable cases were excluded. From the sample (1123), 20 ungradable cases were excluded for the analysis, and 219 cases were categorized as positive.

Using the same threshold as in the original scenario, criterion B produced a sensitivity of 0.945, a specificity of 0.727, and an AUC of 0.918. Criterion C produced a sensitivity of 0.963, a specificity of 0.743, and an AUC of 0.924.

Analyzing the FN rate by severity level (excluding ungradable exams), the results were as follows: 3.65% FN rate for R1 or greater (and/or suspicion DME), 3.54% for R2 or greater (and/or suspicion DME), 4.27% for R3 or greater (and/or suspicion DME), and 5.38% for R4 or greater (and/or suspicion DME).

## Discussion and conclusion

As established in the literature, the minimum sensitivity for screening tests with a dichotomous outcome should be set at 0.8 [28, 29]. A specificity of 0.5 was defined by the commission of specialists and has been shown to be cost-effective [32, 33]. The operating point chosen on the ROC curve produced a sensitivity of 0.946 (95% CI, 0.909–0.969) and specificity of 0.743 (95% CI, 0.733–0.750), with an AUC of 0.915. These results exceeded the performance requirements solicited by Minsal.

The reported sensitivity and specificity of the assessed tool could free up reading capacities in an estimated 59.7% (see Table 3). These released resources can be expected to be relocated to increasing today’s low DR screening coverage (and pursue its periodicity, which will also make up for possible FN), reading additional scans, and treating DR cases that require ophthalmologist care. This point is highly relevant in Chile, given that the public health system guarantees coverage for DM and DR diagnosis and treatment.

The results using different criteria for considering a case as positive show similar sensitivity and specificity levels across the three analyzed scenarios. This illustrates how an AI-based system can outperform the baseline scenario in a sample that faithfully represents the local population. If different screening criteria or guidelines are needed in the future, the threshold of the AI system could be modified to satisfy the sensitivity and specificity defined by a certain strategy.

The international literature describes similar screening procedures for more than mild non-proliferative DR detection. Among the most recent published findings using publicly available databases, one system was found to show a sensitivity of 96.8% and specificity of 59.4% [9], and another one showed a sensitivity of 93.8% and specificity of 72.2% [34]. A study carried out at the Moorfields Reading Centre using data from the population enrolled in the Nakuru Study in Kenya reported a sensitivity of 91.0% and specificity of 69.9% [11]. Another group proposed two automated methods: a disease-staging system for DR and a system that suggests treatments and prognosis. These authors reported an accuracy of 81% for the first system and a 12% FN and 65% FP rate for the second [35]. A recent publication described an automated system that detects R3 and R4 cases with a sensitivity of 66.4% and specificity of 72.8% [29]. Another method achieved over 96% sensitivity and 93% specificity using public datasets, deeming further research necessary in terms of applicability in a clinical setting [10]. Finally, other authors reported a sensitivity of 87.2% and specificity of 90.7% [36].

It should be noted that the quality of ocular fundus photography is typically lower in an older population, such as the one sampled in this study, as patient age and image clarity are inversely correlated in teleophthalmology [37]. Therefore, a relatively high percentage of patients may be referred to a specialist due to unreadable exams.

The effect of the camera model could not be measured and separated from the technician effect at each point of care because of the centres’ sample size. It is proposed for a future study to design the setup so that the effect of a certain camera model and specifications can be measured and separated from the centre and the possible bias of the professional who operates the camera. Another limitation was the data collection related to the procedure performed by the technician, particularly about the mydriasis in each patient, an issue that can affect the image quality and, consequently, the cost-effectiveness of the screening.

Despite the study being designed according to the Minsal screening programme and its standard of care, it is proposed for a future study to assess each case by more than one physician to mitigate the bias related to the eye specialist, as other studies have reported [10, 38].

This system’s implementation is feasible for healthcare workers to carry on given that the interface for the tool is based on the standard teleophthalmology processes already in use according to the Minsal protocol. Moreover, this technology is suitable for application throughout the country as it was developed in collaboration with Minsal, plus regional guidelines, and exceeds the required indicators. Implementation of this AI-based tool is also viable in other countries facing similar challenges in terms of DR screening coverage.

Further analysis should be carried out to determine specific figures in terms of savings and impact, especially considering a real clinical setting and variability in implementation conditions. Future research should study and build a monitoring method for the technology’s performance. Nonetheless, current test negativity rate-estimated freed-up resources would widely compensate for FP rechecking.

Finally, it should be noted that while previous studies established the efficacy and efficiency of tools similar to the one being validated through this study [32, 33], the feasibility of deploying this type of system in the clinical setting had yet to be demonstrated [10, 39]. Due to a combination of clinical and research efforts, this study has shown how an automated tool might be integrated with an existing healthcare system, representing a milestone of collaboration and technological development that can be expected to meaningfully improve Chilean public health. This clinical validation illustrates the potential benefits of AI in addressing global health challenges, providing an example that might be replicated in other developing countries.

## Summary

### What was known before

- Interventions on patients with diabetes: there is a need for more cost-effective interventions to screen diabetic patients for DR—Technology and methods for validation: artificial intelligence-based systems can achieve great precision in DR detection (in controlled settings).

### What this study adds

- Technology and methods for validation: the study reports evidence of an artificial intelligence-based screening tool validated in a clinical setting—Interventions on patients with diabetes: the study reports evidence of validation in a clinical setting agreed with a national health system for its deployment.

## References

[1] International Diabetes Federation. *IDF* Diabetes Atlas Eighth Edition 2017. Brussels, Belgium: International Diabetes Federation; 2017.

[2] International Diabetes Federation, The Fred Hollows Foundation. Diabetes eye health: A guide for health care professionals. Brussels, Belgium: International Diabetes Federation, The Fred Hollows Foundation; 2015.

[3] Wilson JMG, Jungner G. Principles and practice of screening for disease. Geneva: World Health Organization; 1968.

[4] Commission on Chronic Illness. Chronic Illness in the United States: Volume I. Prevention of chronic illness, Vol. 1. Harvard University Press: Cambridge, Massachusetts; 1957.

[5] Barría F, Martínez F, Verdaguer J. Actualización de la Guía clínica de Retinopatía Diabética para Latinoamérica. Vision*.* 2020 2016: 1–26.

[6] Resnikoff S, Felch W, Gauthier T-M, Spivey B (2012). The number of ophthalmologists in practice and training worldwide: a growing gap despite more than 200 000 practitioners. Br J Ophthalmol.

[7] Schlottmann P, Acosta C, Alezzandrini A, Bafalluy J, Biccas L, Cano RH (2014). Defining Best Practice Standards for the Diagnosis and Management of Diabetic Retinopathy and Diabetic Macular Edema in Latin America. Vis Pan-Am, Pan-Am J Ophthalmol.

[8] International Council of Ophthalmology. ICO Guidelines For Diabetic Eye Care. 2017. http://www.icoph.org/downloads/ICOGuidelinesforDiabeticEyeCare.pdf. Accessed 7 August 2020.

[9] Abràmoff MD, Folk JC, Han DP, Walker JD, Williams DF, Russell SR (2013). Automated analysis of retinal images for detection of referable diabetic retinopathy. JAMA Ophthalmol.

[10] Gulshan V, Peng L, Coram M, Stumpe MC, Wu D, Narayanaswamy A (2016). Development and validation of a deep learning algorithm for detection of diabetic retinopathy in retinal fundus photographs. Jama.

[11] Hansen MB, Abràmoff MD, Folk JC, Mathenge W, Bastawrous A, Peto T (2015). Results of automated retinal image analysis for detection of diabetic retinopathy from the Nakuru study, Kenya. PloS ONE.

[12] Kapetanakis VV, Rudnicka AR, Liew G, Owen CG, Lee A, Louw V (2015). A study of whether automated Diabetic Retinopathy Image Assessment could replace manual grading steps in the English National Screening Programme. J Med Screen.

[13] International Diabetes Federation, International Council of Ophthalmology, World Council of Optometry, International Agency for the Prevention of Blindness. Strengthening health systems to manage diabetic eye disease: Integrated care for diabetes and eye health. International Diabetes Federation, International Council of Ophthalmology, World Council of Optometry, International Agency for the Prevention of Blindness; 2017. https://www.idf.org/news/99:idf-and-leading-eye-health-organizations-call-for-urgent-action-to-address-diabetic-eye-disease.html. Accessed 7 August 2020.

[14] Ministerio de Salud de Chile. Encuesta Nacional de Salud 2003. Santiago, Chile: Ministerio de Salud; 2004.

[15] Ministerio de Salud de Chile. Servicios de Salud - Ministerio de Salud - Gobierno de Chile. https://www.minsal.cl/servicios-de-salud/. Accessed 7 August, 2020.

[16] Ministerio de Salud de Chile. Redes de atención GES y no GES. https://auge.minsal.cl/website/doc/redes-ges-y-no-ges-2020.pdf. Accessed 7 August, 2020.

[17] Ministerio de Salud de Chile. Guía de Práctica Clínica - Problema de Salud AUGE N°31, Retinopatía Diabética. Santiago, Chile: Ministerio de Salud; 2017.

[18] Arenas-Cavalli JT (2013). Automated diabetic retinopathy detection based on remote computational intelligence. IEEE e-Health Tech Comm.

[19] Donoso R, Arenas-Cavalli JT, Pola M. Validación de un sistema de screening automatizado de retinopatía diabética en un centro de referencia de salud público en Chile. XXI Brazilian Congress of Ophthalmology, Blindness Prevention and Visual Rehabilitation; Recife, Brazil, 2014.

[20] Arenas-Cavalli JT, Ríos SA, Pola M, Donoso R (2015). A web-based platform for automated diabetic retinopathy screening. Procedia Computer Sci.

[21] Wilkinson C, Ferris FL, Klein RE, Lee PP, Agardh CD, Davis M (2003). Proposed international clinical diabetic retinopathy and diabetic macular edema disease severity scales. Ophthalmology.

[22] Aldington S, Kohner E, Meuer S, Klein R, Sjølie A, Group EICS (1995). Methodology for retinal photography and assessment of diabetic retinopathy: the EURODIAB IDDM complications study. Diabetologia.

[23] Abràmoff MD, Reinhardt JM, Russell SR, Folk JC, Mahajan VB, Niemeijer M (2010). Automated early detection of diabetic retinopathy. Ophthalmology.

[24] Graham C. Anonymisation: managing data protection risk code of practice. Information Commissioner’s Office; 2012. https://ico.org.uk/media/1061/anonymisation-code.pdf. Accessed 7 August 2020.

[25] De Fauw J, Keane P, Tomasev N, Visentin D, van den Driessche G, Johnson M (2016). Automated analysis of retinal imaging using machine learning techniques for computer vision. F1000Research.

[26] General Assembly of the World Medical Association. (2014). World Medical Association Declaration of Helsinki: ethical principles for medical research involving human subjects. J Am Coll Dent.

[27] Long J, Shelhamer E, Darrell T. Fully convolutional networks for semantic segmentation. Proc IEEE Conf Comput Vis Pattern Recogn. 2015: 3431–3440. https://www.cv-foundation.org/openaccess/content_cvpr_2015/html/Long_Fully_Convolutional_Networks_2015_CVPR_paper.html. 10.1109/TPAMI.2016.2572683 27244717

[28] British Diabetic Association. Retinal photography screening for diabetic eye disease. BDA: London; 1997.

[29] Walton OB, Garoon RB, Weng CY, Gross J, Young AK, Camero KA (2016). Evaluation of automated teleretinal screening program for diabetic retinopathy. JAMA Ophthalmol.

[30] Bouhaimed M, Gibbins R, Owens D (2008). Automated detection of diabetic retinopathy: results of a screening study. Diabetes Technol Ther.

[31] Xie Y, Gunasekeran DV, Balaskas K, Keane PA, Sim DA, Bachmann LM (2020). Health Economic and Safety Considerations for Artificial Intelligence Applications in Diabetic Retinopathy Screening. Transl Vis Sci Technol.

[32] Tufail A, Kapetanakis VV, Salas-Vega S, Egan C, Rudisill C, Owen CG (2016). An observational study to assess if automated diabetic retinopathy image assessment software can replace one or more steps of manual imaging grading and to determine their cost-effectiveness. Health Technol Assess.

[33] Tufail A, Rudisill C, Egan C, Kapetanakis VV, Salas-Vega S, Owen CG (2017). Automated diabetic retinopathy image assessment software: diagnostic accuracy and cost-effectiveness compared with human graders. Ophthalmology.

[34] Solanki K, Ramachandra C, Bhat S, Bhaskaranand M, Nittala MG, Sadda SR (2015). EyeArt: automated, high-throughput, image analysis for diabetic retinopathy screening. Investig Ophthalmol Vis Sci.

[35] Takahashi H, Tampo H, Arai Y, Inoue Y, Kawashima H (2017). Applying artificial intelligence to disease staging: deep learning for improved staging of diabetic retinopathy. PloS ONE.

[36] Abràmoff MD, Lavin PT, Birch M, Shah N, Folk JC (2018). Pivotal trial of an autonomous AI-based diagnostic system for detection of diabetic retinopathy in primary care offices. NPJ Digital Med.

[37] Scanlon PH, Foy C, Malhotra R, Aldington SJ (2005). The influence of age, duration of diabetes, cataract, and pupil size on image quality in digital photographic retinal screening. Diabetes Care.

[38] Krause J, Gulshan V, Rahimy E, Karth P, Widner K, Corrado GS (2018). Grader variability and the importance of reference standards for evaluating machine learning models for diabetic retinopathy. Ophthalmology.

[39] Beede E, Baylor E, Hersch F, Iurchenko A, Wilcox L, Ruamviboonsuk P et al. A Human-Centered Evaluation of a Deep Learning System Deployed in Clinics for the Detection of Diabetic Retinopathy. Proc 2020 CHI Conf Human Factors Comput Syst. 2020:1–12. https://dl.acm.org/doi/abs/10.1145/3313831.3376718.

