# Correction: Clinical validation of an artificial intelligence-based diabetic retinopathy screening tool for a national health system

**DOI:** 10.1038/s41433-021-01690-z

**Published:** 2021-07-23

**Authors:** José Tomás Arenas-Cavalli, Ignacio Abarca, Maximiliano Rojas-Contreras, Fernando Bernuy, Rodrigo Donoso

**Affiliations:** 1 TeleDx, Santiago, Chile; 2 Department of Ophthalmology, Universidad de Chile, Santiago, Chile

**Keywords:** Public health, Retinal diseases

Correction to: *Eye* 10.1038/s41433-020-01366-0, published online 11 January 2021

Unfortunately, an error occurred in Table 3; the formula for specificity should be read as sp = TN/TN + FP.

The original article has been corrected.

